# *Bartonella quintana*, an Unrecognized Cause of Infective Endocarditis in Children in Ethiopia

**DOI:** 10.3201/eid2308.161037

**Published:** 2017-08

**Authors:** Diana Tasher, Alona Raucher-Sternfeld, Akiva Tamir, Michael Giladi, Eli Somekh

**Affiliations:** Tel Aviv University Sackler School of Medicine, Tel Aviv, Israel (D. Tasher, A. Raucher-Sternfeld, A. Tamir, M. Giladi, E. Somekh);; Wolfson Medical Center, Holon, Israel (D. Tasher, A. Raucher-Sternfeld, A. Tamir, E. Somekh);; Tel Aviv Sourasky Medical Center, Tel Aviv (M. Giladi);; European Paediatric Association–Union of National European Paediatric Societies and Associations, Berlin, Germany (E. Somekh)

**Keywords:** Bartonella quintana, Bartonella spp., children, infective endocarditis, heart defects, heart failure, culture-negative endocarditis, East Africa, Ethiopia, Israel, Germany, bacteria, vector-borne infections, lice, parasites

## Abstract

*Bartonella quintana* endocarditis, a common cause of culture-negative endocarditis in adults, has rarely been reported in children. We describe 5 patients 7–16 years of age from Ethiopia with heart defects and endocarditis; 4 cases were caused by infection with *B. quintana* and 1 by *Bartonella* of undetermined species. All 5 patients were afebrile and oligosymptomatic, although 3 had heart failure. C-reactive protein was normal or slightly elevated, and erythrocyte sedimentation rate was high. The diagnosis was confirmed by echocardiographic demonstration of vegetations, the presence of high *Bartonella* IgG titers, and identification of *B. quintana* DNA in excised vegetations. Embolic events were diagnosed in 2 patients. Our data suggest that *B. quintana* is not an uncommon cause of native valve endocarditis in children in Ethiopia with heart defects and that possible *B. quintana* infection should be suspected and pursued among residents of and immigrants from East Africa, including Ethiopia, with culture-negative endocarditis.

## Abstract 2

Infection is probably not uncommon in those with heart defects, and diagnosis should be considered for patients with culture-negative endocarditis.

Blood culture–negative endocarditis accounts for 2.5%–31% of all cases of endocarditis (*1*). *Bartonella* spp. (most commonly *B. henselae* and *B. quintana*) are among the most common causes of blood culture–negative endocarditis, being responsible for 9.5%–28.4% of all cases (*2*,*3*). The literature regarding* Bartonella* endocarditis among children describes 1 microbiologically confirmed case caused by *B. quintana* (*4*) and 8 cases caused by *B. henselae* (*5*–*12*). In this case series, we describe 5 patients, 7–16 years of age, from Ethiopia who were referred to our center in Israel for heart surgery and diagnosed with endocarditis caused by *B. quintana* (4 cases) or *Bartonella* of an undetermined species (1 case).

## Materials and Methods

Wolfson Medical Center in Holon, Israel, provides cardiothoracic care to children from developing regions, such as Africa, Palestinian Authority, and South America, who are referred for care through the Save a Child’s Heart fund activity (*13*). Each year, doctors in the Center perform heart surgery on ≈200 children from developing countries. After the unexpected diagnosis of *B. quintana* endocarditis in this group of children, we reviewed the presurgical cases of infective endocarditis in children referred for heart surgery during 2006–2015. 

Before surgery, we conducted a thorough work-up on all patients with endocarditis, including several blood cultures and serologic testing for *Bartonella,* *Legionella*, and *Brucella* spp. and for Q fever (*Coxiella burnetii* infection). In addition, for most patients, we performed microbiologic studies of specimens obtained during cardiac surgery. We performed and interpreted enzyme immunoassays that have been shown to be 98% specific for detection of *B. henselae* IgM and IgG (*14*,*15*). We also performed serial dilutions to determine final serum titers. 

Similar to other serologic assays used for the diagnosis of *Bartonella* infections (*16*,*17*), the enzyme immunoassay used in this study demonstrated high cross-reactivity between *B. quintana* and *B. henselae* (M. Giladi, unpub. data). Thus, we presented serologic results as *Bartonella* sp. IgG and IgM, without species identification. We also performed a genus-specific amplification assay of a 588-bp fragment of the *Bartonella* riboflavin synthase gene as previously reported (*18*). For species identification, we sequenced the PCR product and submitted it to a BLAST search (https://blast.ncbi.nlm.nih.gov/Blast.cgi). We considered patients to have endocarditis only after they were determined to fulfill the Duke criteria (*19*).

## Results

From January 1, 2006, through December 31, 2015, a total of 574 children from Africa underwent cardiac surgery, catheterization, or both at Wolfson Medical Center. During this time, 7 (1.2%) of the 574 children were diagnosed with infective endocarditis before surgery. The most frequent cause of endocarditis was *B. quintana* infection, which was diagnosed in 4/7 children. The 3 other endocarditis cases were caused by undetermined *Bartonella* spp. infection in an 11-year-old boy from Ethiopia, *Streptococcus viridans* infection in a 2-year-old boy from Zanzibar, and Q fever in a 7-year-old girl from Kenya. All patients with *Bartonella* endocarditis were from Ethiopia, where they lived in poor, crowded conditions. The clinical details of the children with *Bartonella* endocarditis follow.

### Cases

#### Patient A

An asymptomatic 7-year-old girl from Jimma, Ethiopia, with patent ductus arteriosus was referred to the Wolfson Medical Center. At admission, she was afebrile and in good general condition. Her physical examination revealed a grade 2/6 continuous cardiac murmur and splenomegaly but no stigmata of infective endocarditis. Transthoracic echocardiogram (TTE) revealed thickening of the pulmonary valve and the presence of a large (1.1 cm in diameter) mobile mass attached to the middle of the main pulmonary artery and several small masses attached to the pulmonic valve cusps, consistent with vegetations. The main laboratory results were a high erythrocyte sedimentation rate (ESR) and an only slightly elevated CRP (Table).

**Table:** Features for children from Ethiopia with *Bartonella quintana* endocarditis*

Patient, age, y/sex	Echo finding	Underlying condition	Phenomena		Microbiologic evidence	CRP, mg/dL	ESR, mm/h	Leuk/mm^3^	Hb, g/dL	Platelets/ mm^3^†
			Vascular	Immunologic						
A, 7/F	Several vegetations (11 mm) on pulmonary valve	CHD	No	RF Ab, 189 IU/mL	*Bartonella* IgG titer 1:1,600, IgM neg; excised vegetation PCR-neg for *Coxiella burnetii*, PCR-pos for *B. quintana*‡	1.7	128	3,500	8.5	150,000
B, 12/F	Vegetation (7 mm) on mitral valve	RHD	No	RF Ab, <10 IU/mL	*Bartonella* IgG titer 1:100, IgM neg; excised vegetation PCR-neg for *C. burnetii*, PCR-pos for *B. quintana*‡	0.7	50	5,700	10.3	310,000
C, 16/F	Two vegetations (5 mm) on aortic valve	RHD	Emboli to spleen	GN; 16 Osler nodes	*Bartonella* IgG titer 1:12,800 (10 wks after surgery: 1:6,400); excised vegetation PCR-neg for *C. burnetii*, PCR-pos for *B. quintana*‡	4.2	150	6,900	7.8	334,000
D, 9/F	Several vegetations (10 mm and 20 mm) on aortic valve	CHD	CVA	RF Ab, 25 IU/mL	*Bartonella* IgG titer 1:6,400 (5 wks after surgery: 1:1,600); excised vegetation PCR-neg for *C. burnetii*, PCR-pos for *B. quintana*‡	1.5	44	6,100	11.7	189,000
E, 11/M	Vegetation (14 mm) on aortic valve	CHD	No	RF Ab, 2,560 IU/mL	*Bartonella* IgG titer 1:6,400; *C. burnetii* IgM neg	2.0	110	9,100	11.8	264,000

*HIV serologic testing and blood culture results were negative for all patients, and no patients had fever. Ab, antibody; CHD, congenital heart disease CRP, C-reactive protein (reference range 0–10 mg/dL); CVA, cerebral vascular accident; Echo, echocardiogram; ESR, erythrocyte sedimentation rate (reference range 0–20 mm); GN, glomerulonephritis; Hb, hemoglobin (reference range 9.5–13.0 g/dL); Leuk, leukocytes (reference range 6,000–17,500 cells/mm^3^); neg, negative; pos, positive; RF, rheumatoid factor; RHD, rheumatic heart disease. †Reference range 150–400 × 10^9^ platelets/L. ‡Sequence comparison analysis demonstrated a 100% identity to *B. quintana*.

We obtained blood samples for cultures and then initiated empirical therapy with doxycycline and ceftriaxone for presumed common and culture-negative endocarditis pathogens. The serologic test result for *Bartonella* spp. was positive (IgG titer of 1:1,600), but the test result for IgM was negative (Table). Thus, we initiated treatment with intravenous gentamicin (3 mg/kg bodyweight 1×/d) and oral doxycycline (2 mg/kg bodyweight every 12 h) for *Bartonella* endocarditis (*20*,*21*). After 2 weeks of treatment, the patient underwent surgical repair of her heart defect. The vegetations were excised from the main pulmonary artery and the pulmonic valve cusps, a perforation that was revealed in the right pulmonary valve cusp was repaired, and the patent ductus arteriosus was closed. The child had an uneventful postoperative course. PCR testing confirmed the presence of *B. quintana* DNA in the excised vegetation. The child completed a 2-week course of gentamicin and a 5-week course of oral doxycycline and returned to Ethiopia in good condition.

#### Patient B

A 12-year-old girl from Jimma was admitted for the correction of mitral regurgitation due to rheumatic heart disease, which had been diagnosed 4 months earlier due to symptoms of congestive heart failure. On physical examination, she was afebrile and exhibited tachypnea and orthopnea. Cardiac examination revealed a grade 4/6 harsh systolic ejection murmur and a grade 2/4 diastolic rumble at the apex. Physical findings were otherwise unremarkable and showed no stigmata of infective endocarditis. TTE revealed the presence of a 7-mm mobile mass, consistent with vegetation, attached to the mitral chordae. Main laboratory results were high erythrocyte sedimentation rate (ESR) and an only slightly elevated CRP (Table).

We initiated empirical therapy with doxycycline and ceftriaxone, and after serologic results were received, we initiated treatment with gentamicin and oral doxycycline for *Bartonella* endocarditis. After 2 weeks of treatment with doxycycline, the child underwent mitral valvuloplasty; she had an uneventful postoperative course. PCR testing confirmed the presence of *B. quintana* DNA in the excised vegetations. The child completed a 6-week course of oral doxycycline and a 1-week course of gentamicin (during the first week of doxycycline treatment) and returned to Ethiopia in good condition.

#### Patient C

A 16-year-old girl from Jimma was admitted for surgical repair of mitral and aortic valve disease due to rheumatic heart disease that had been diagnosed when she was 12 years of age. During the previous year, she had had several heart failure–related hospitalizations. At admission, she was afebrile and exhibited orthopnea and dyspnea. Cardiac examination revealed a grade 3/4 diastolic murmur, and abdominal examination showed liver enlargement. Physical findings were otherwise unremarkable, with no stigmata of infective endocarditis. TTE revealed 2 vegetations (5-mm diameter) attached to the noncoronary aortic valve cusp. As with the previous patients, her laboratory results showed high ESR and only slightly elevated CRP (Table).

After receiving the serologic test results, we initiated treatment with gentamicin and doxycycline. After 2 weeks of treatment, painful reddish lesions suggestive of Osler nodes developed on the patient’s palms, and her spleen was enlarged 4 cm below the costal margin. Repeated TTE revealed a single vegetation. Abdominal ultrasonography showed enlarged spleen (14.5 cm in length) with infarcts compatible with emboli. The patient underwent surgical repair of her mitral and aortic valves. PCR testing confirmed the presence of *B. quintana* DNA in the excised vegetation. The patient completed a 12-week course of oral doxycycline and a 2-week course of gentamicin (during the first 2 weeks of doxycycline treatment). Six months after surgery she was asymptomatic.

#### Patient D

A 9-year-old girl from Addis Ababa, Ethiopia, was referred for surgical repair of a congenital heart defect. One year before admission, she had a history of febrile illness accompanied by left-sided weakness. The patient was diagnosed in Ethiopia with a large patent ductus arteriosus and severe aortic regurgitation and was suspected to have infective endocarditis. At admission, she was afebrile but had systolic and diastolic murmurs and hepatosplenomegaly. Neurologic examination revealed left hemiparesis. She had no other stigmata of infective endocarditis. TTE revealed several calcified vegetations (10 mm and 20 mm in diameter) attached to the cusps of the aortic valve. 

We initiated treatment with gentamicin and oral doxycycline. After receiving treatment for a week, the patient underwent aortic valve replacement, mitral valve repair, and patent ductus arteriosus closure. PCR of the excised aortic valve revealed *B. quintana* DNA. The child had an uneventful postoperative course. She completed a 2-week course of gentamicin and a 10-week course of oral doxycycline.

#### Patient E

An 11-year-old boy from Addis Ababa was referred for repair of a large coronary artery fistula. He complained mainly of weakness on exertion and chest pain. Physical examination revealed unremarkable vital signs and temperature, continuous machinery murmur, and splenomegaly but no rash or endocarditis stigmata. An echocardiograph revealed a 14-mm vegetation on the aortic valve. Serologic studies revealed a high IgG titer (1:6,400) to *Bartonella* sp.; IgM results were negative. The results of other serologic studies and multiple blood cultures were negative (Table).

We initiated treatment with gentamicin (for 2 weeks) and oral doxycycline (for 3 months). The heart defect was corrected by catheterization without surgery, so we did not have tissue for molecular studies and could not determine the *Bartonella* species. We followed the child for 5 months in our center; he was asymptomatic, and repeated echocardiography showed gradual regression of the vegetation size until its actual disappearance.

## Discussion

This case series provides detailed information regarding the clinical presentation, course, and outcome of *Bartonella* endocarditis caused by *B. quintana* infection in 4 children and by *Bartonella* of undetermined species in 1 child. Of interest, all 5 children were natives of Ethiopia. When admitted to our medical center, all of the children were afebrile and had nonspecific symptoms, except for heart failure, which was attributed to their previously known heart disease. In 4 of the 5 patients, endocarditis was not suspected on clinical grounds, but we pursued the diagnosis after echocardiographs revealed vegetations. The diagnosis of *B. quintana* endocarditis was confirmed in 4 patients (patients A–D) by identification of *B. quintana* DNA in excised vegetations or endocardial tissue. The diagnosis was further supported by the presence of *Bartonella* IgG in these 4 patients, 3 of whom had high titers (1:1,600–1:12,800). The fifth patient had *Bartonella* endocarditis caused by an undetermined species; the diagnosis was based only on serologic test results (IgG titer of 1:6,400) because cardiac tissue was not available for molecular diagnosis. Western blot with cross-absorption studies, a method described by Houpikian and Raoult (*22*), could have discriminated between *B. quintana* and *B. henselae* if it had been applied.

*Bartonella* spp. are small, gram-negative bacilli whose natural cycle includes a reservoir host, in which *Bartonella* causes chronic intraerythrocytic bacteremia. In 1993, *Bartonella* spp. were described as a cause of endocarditis in 2 separate reports and subsequently has become appreciated as a substantial cause of culture-negative endocarditis (*3*,*23*,*24*). Raoult and colleagues have generated several reports on endocarditis caused by *Bartonella* spp., including several multicenter international studies that involved patients from France, England, and Canada (*17*,*25*). Seven *Bartonella* spp. have been reported to cause infective endocarditis in humans; >95 percent of the cases involved *B. quintana* or *B. henselae* (*17*).

This case series of *Bartonella* endocarditis in children reveals several common characteristics. All cases occurred in preadolescent and adolescent patients; all patients were afebrile, and the main pathophysiologic dysfunction was congestive heart failure. All patients had markedly elevated ESRs but normal or only mildly elevated CRP levels. Echocardiography revealed large and even giant vegetations in most of the patients, and 2 (40%) of the patients had embolic phenomena. Medical treatment consisted of a prolonged course of doxycycline combined with gentamicin during the initial period, as was recommended for adults with *Bartonella* endocarditis (*21*). Even though prolonged administration of doxycycline is relatively contraindicated in children <8 years of age, we suggested a 5-week course for the 7-year-old patient (patient A), as recently recommended (*20*), because of the extent of her valvular disease.

*B. quintana* is historically known to cause trench fever, a recurrent febrile disease with acute onset characterized by fever and headache. Trench fever was epidemic among troops during World War I, causing millions of casualties. However, after the introduction of louse control measures, the disease was no longer considered a threat. Recently, however, trench fever has reemerged, causing bacteremia in homeless persons and persons affected with alcoholism in Europe and North America, where it has now been designated urban trench fever (*26*). *B. quintana* is mostly associated with human body lice but has also been found in fleas (*27*,*28*). The predisposing factors for *B. quintana* endocarditis are homelessness, alcoholism, and exposure to body lice (*29*). None of these risk factors for *B. quintana* infection were known to exist in the patients in this study.

Patients in this study denied having had lice infestation in the past, and we did not identify body lice, pruritus, or excoriations during the initial physical examinations. However, we believe that detailed and accurate histories regarding lice infestation were lacking, particularly because patients with *B. quintana *endocarditis have probably been infected with *B. quintana* for months or years before hospital admission for endocarditis. We speculate that residence in a developing country with presumably poor hygiene and low socioeconomic status might have exposed the patients in this study to ectoparasite infestations, including body lice, which could have served as a transmitting vector for *B. quintana*.

In contrast to our report of afebrile patients with sizable vegetations, previous reports of *Bartonella* endocarditis have described that fever is usually present (83% of cases) and that valve destruction is characterized by large calcifications but small vegetations (*25*). Description of *B. quintana* endocarditis in children is currently confined to a case in a 13-year-old girl from Senegal with underlying rheumatic heart disease, an insidious afebrile clinical course, and prominent vegetations of the left side of the heart (*4*). Another 2 children with endocarditis and *B. quintana*–positive serologic test results were included in a series from India, but no clinical or laboratory details were provided (*30*).

Of the 5 children in our study with *Bartonella* endocarditis, 4 had involvement of the aortic valve. The predilection for *Bartonella* spp. to infect the aortic valve has been described (*25*), but the reason is unknown. Before surgery, 4 of the 5 children fulfilled the Duke criteria for definite infective endocarditis, and the fifth child (patient E) fulfilled criteria for possible endocarditis (Table).

The Duke criteria do not address *Bartonella* endocarditis specifically, and a definitive diagnosis of *Bartonella* infection requires positive, high-titer serologic test results; PCR identification of *Bartonella* sp. DNA in affected tissue or blood; or, on rare occasions, isolation of *Bartonella* sp. from blood or tissue culture. Recent studies have shown that direct immunofluorescence antibody assays can reliably detect *Bartonella* IgG, and an IgG titer of >1:800 has a high positive predictive value (95.5%) for *Bartonella* infection among patients with endocarditis (*31*,*32*). However, in 2015, Edouard et al. (*32*) reported that an IgG titer of <800 does not exclude the diagnosis of *Bartonella* endocarditis in patients with valvulopathy and that a serologic diagnosis can be confirmed by a positive Western blot result, which they showed exhibited a sensitivity of 100%. Similarly, we showed that high *Bartonella* IgG titers can be detected by enzyme immunoassay; only 1 patient in our series had IgG titers <1:800. Thus, the enzyme immunoassay has a meaningful role in the diagnosis of *Bartonella* sp. endocarditis.

Epidemiologic data suggest a north–south gradient distribution in the prevalence of *Bartonella* endocarditis, from 0% in Sweden to 3% in France and Germany and reaching 15.6% in Algeria and 9.8% in Tunisia (*32*). Lice are a well-recognized reservoir of *B. quintana*. Using reverse transcription PCR testing of lice from residents of 9 African countries, Sangaré et al. (*33*) showed *B. quintana* DNA was present in 54% of body and 2% of head lice, and they found a clear correlation between the presence of *B. quintana* in head and body lice and the degree of country poverty, as determined by the gross domestic product. *Bartonella* spp. were found among 6 (9.2%) of 65 head lice pools and 1 (3.0%) of 33 clothing lice pools from Jimma (*34*). These data indicate that *B. quintana* may be quite abundant in East Africa. However, due to the lack of serologic surveys for *Bartonella* species in this region, its extent is unknown. 

In our series, *B. quintana* was the most frequent causative organism of native valve endocarditis among children from Africa referred to our center for heart surgery. These cases by no means represent the whole spectrum of infective endocarditis in children in Africa, or even Ethiopia, because a selection bias might exist toward cases of nonacute, indolent, infective endocarditis in patients referred for complicated surgeries. However, the predominance of *B. quintana* infection, even in this specific, small subgroup of patients, is quite impressive and may imply a broader role of this microorganism in infective endocarditis cases in children in Ethiopia or Africa as a whole.

In conclusion, *B. quintana* is a substantial cause of endocarditis in children in Ethiopia with heart disease. Diagnosis may easily be missed because of the afebrile, insidious nature of this disease and the apparent lack of traditional risk factors for *Bartonella* infections.
